# Mississippi Valley Dental Association

**Published:** 1866-03

**Authors:** 


### MISSISSIPPI VALLEY DENTAL ASSOCIATION.

The 21st annual meeting of this association was held on the 21st, 22nd and 23rd of February. It was by far the largest, and we think in some respects the most interesting and enthusiastic meeting of this society. There was a much larger number of the profession present than ever before, and a very large increase of membership was the result.

The discussions were regarded as highly instructive, they were fully reported, and will be published in the next number of the Register. Several prizes were offered for essays improvements &c. for which see official minutes which will be published.

This society retains its youthful vigor, and will not soon be superceeded by any thing else. It has passed through some severe struggles, and these are some of the things, that make it dear to those who where wont to meet in years by-gone in its councils, and for the future we feel well assured, that when the time for its annual convocation arrives, there will still greater numbers come up for concourse and mutual benefit. We hope to see the profession take hold of the subjects for which prizes are offered, and bring forth something which shall richly merit the award.

